# The immunology of post-kala-azar dermal leishmaniasis (PKDL)

**DOI:** 10.1186/s13071-016-1721-0

**Published:** 2016-08-23

**Authors:** Eduard E. Zijlstra

**Affiliations:** Rotterdam Centre for Tropical Medicine, Bovenstraat 21, 3077 BB Rotterdam, The Netherlands

**Keywords:** Post-kala-azar dermal leishmaniasis, PKDL, Visceral leishmaniasis, Immune responses, Clinical features, Immunosuppression, Immune manipulation

## Abstract

Post-kala-azar dermal leishmaniasis (PKDL) is a common complication of visceral leishmaniasis (VL) caused by *Leishmania donovani*. Because of its possible role in transmission it is considered a public health problem in VL endemic areas. The clinical features include a skin rash consisting of macules, papules or nodules in an otherwise healthy individual; this presentation is determined by the immune response towards parasites in the skin that probably persisted from the previous VL episode. The immune response in VL, cured VL and PKDL is the result of changes in the cytokine profile that only in part can be captured under the Th1 and Th2 dichotomy. Regulatory T cells and Th 17 cells also play a role. VL is characterized by an absent immune response to *Leishmania* with a predominantly Th2 type of response with high levels of IL-10; after successful treatment the patient will be immune with in vitro features of a Th1 type of response and in vivo a positive leishmanin skin test. PKDL takes an intermediate position with a dissociation of the immune response between the skin and the viscera, with a Th2 and Th1 type of response, respectively. It is likely that immune responses determine the different epidemiological and clinical characteristics of PKDL in Asia and Africa; various risk factors for PKDL may influence this, such as incomplete and inadequate treatment of VL, parasite resistance and genetic factors. It should be noted that PKDL is a heterogeneous and dynamic condition and patients differ with regard to time of onset after visceral leishmaniasis (VL), chronicity, extent and appearance of the rash including related immune responses, all of which may vary over time. Better understanding of these immune responses may offer opportunities for manipulation including combined chemotherapy and immunotherapy for VL to prevent PKDL from occurring and similarly in the treatment of chronic or treatment resistant PKDL cases.

## Background

Post-Kala-azar Dermal Leishmaniasis (PKDL) may follow after treatment of visceral leishmaniasis (VL, kala-azar). It is an intermediate disease state before full recovery from VL and is characterized by a skin rash around persisting parasites in the absence of systemic parasitaemia. Alternatively, reinfection after VL may be considered. It is thought that these patients may play a role in transmission of VL [1].

After infection with leishmanial parasites through the bite of the sand fly, individuals may develop VL with fever, hepatosplenomegaly, weight loss and pancytopenia; typically with high levels of antibodies and absent cellular immunity against the *Leishmania* parasites. After successful treatment with antileishmanial drugs the patient becomes immune, as can be demonstrated in vitro by the cellular immune responses and in vivo by the leishmanin skin test (LST) that becomes positive in 80 % of patients [2].

**Fig. 1:**
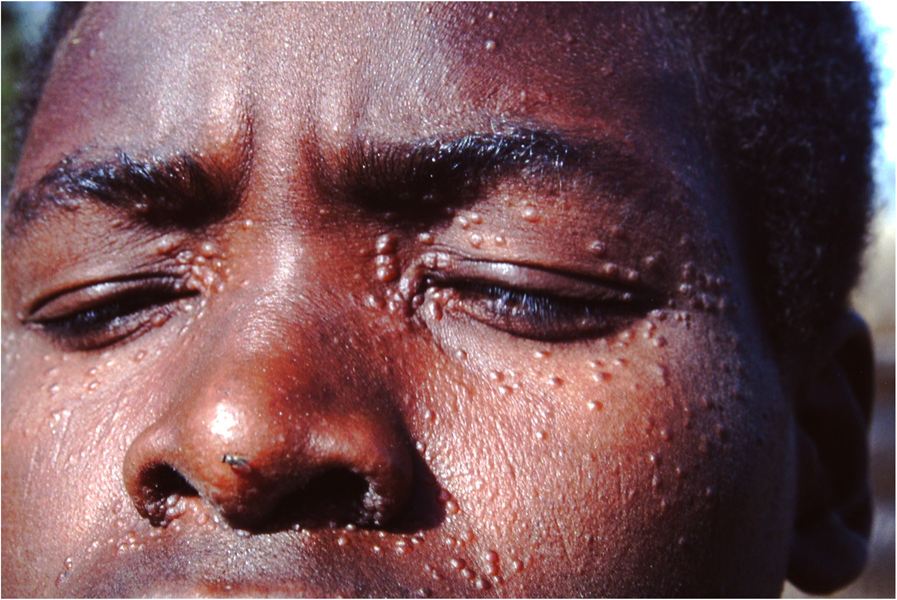
Typical PKDL from Sudan with papular lesions. ©World Health Organization, 2012. Reproduced unmodified from reference [77]

**Fig. 2:**
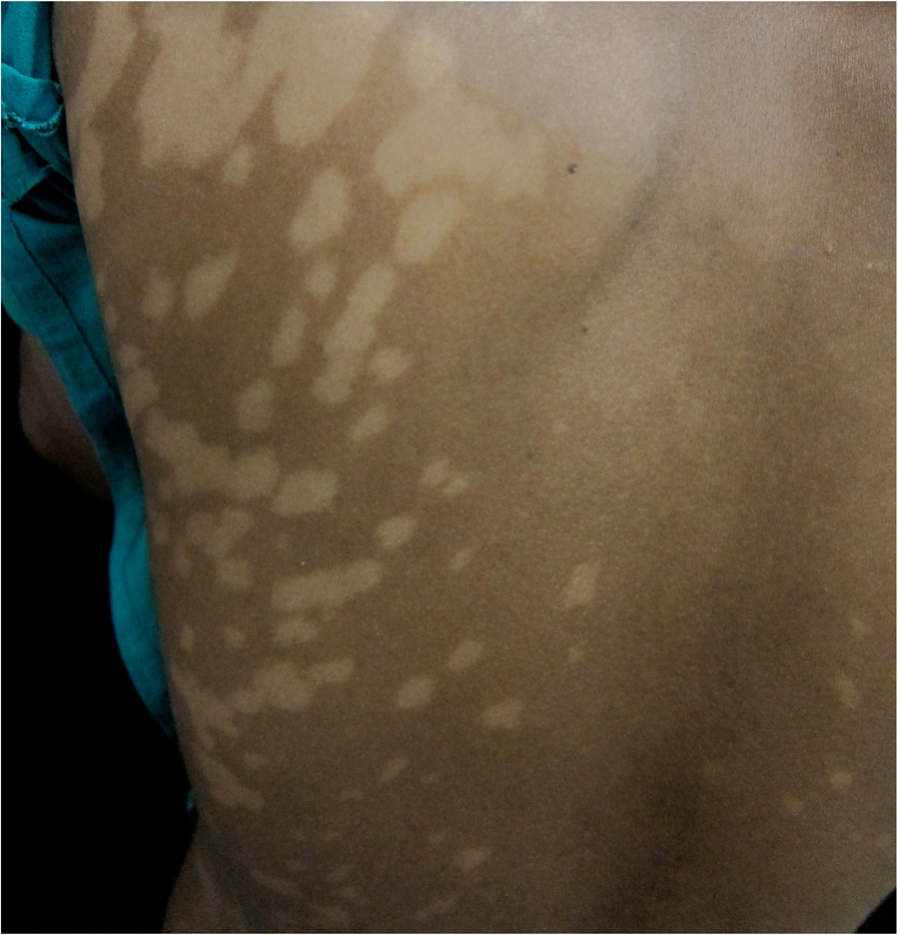
Typical PKDL from Bangladesh with (confluent) macular rash. ©World Health Organization, 2012. Reproduced unmodified from reference [77]

A proportion of treated VL patients may develop PKDL; these patients are not ill, usually do not have hepatosplenomegaly and have recovered from the malnourished state. PKDL is mainly restricted to follow VL caused by *L. donovani*; hence it occurs in Africa and Asia. There are, however, important epidemiological and clinical differences (Table 1). The most striking are the differences in clinical presentation (90 % papular rash in Africa, 90 % macular in Asia) and the interval between VL and PKDL (0–13 months in Africa, usually 2–3 years in Asia) [1, 3], (Figs. 1 and 2). In Africa PKDL is most common in Sudan; most (85 %) patients self-heal within 12 months; only severe or chronic cases are treated [3]. In Asia, although there some reports on self-healing, all patients are treated. After successful treatment with prolonged courses of antileishmanial drugs the rash will disappear and patients will develop immunity similar to cured VL patients. This immunity is permanent unless there is associated immunosuppression.

**Table 1:** PKDL - differences between Africa and Asia

	Asia	Africa
Clinical type most common	macular	papular
Frequency after VL	10–20 %	50–60 %
Infective to sand flies	yes	yes
Transmission of VL	anthroponotic	anthroponotic and zoonotic
Interval after VL	0–3 years, or more^a^	0–13 months
Self-cure	yes, probably	yes
Occurs without previous VL	yes	yes
Occurs with VL simultaneously^b^	yes	yes
Occurs with mucosal involvement	yes	yes
Treatment policy	all are treated	chronic > 6 months or severe PKDL are treated
Treatment	miltefosine	SSG or AmBisome
Marker for cure	clinical	clinical

^a^recent data show that up to 30 % of PKDL in Asia occurs within 12 months after treatment for VL

^b^this is also called para-kala-azar dermal leishmaniasis

Other patients present with VL and PKDL simultaneously (para-kala-azar leishmaniasis); alternatively around 10 % present with PKDL without a previous history of VL. In HIV co-infection, the frequency and clinical presentation of PKDL is different. All these different manifestations strongly suggest a pivotal role for the immune response that is influenced by a number of factors, some of which have been well described; however, there remain important issues to be clarified.

In this paper we describe the principal immunological features of PKDL, in particular in relation to VL, and correlate these findings with clinical and epidemiological knowledge. In addition issues in immunosuppression and immunomodulation will be discussed. Lastly, areas of future research will be outlined.

## Immune responses in VL and PKDL

Immune responses in leishmaniasis are often described in terms of a (T helper) Th1 and Th2 response. This is derived from the murine model of cutaneous leishmaniasis in which a dichotomy was found with a Th2 response during disease and a Th1 response indicating cure [4, 5]. In VL in humans this dichotomy is not clear and shows overlap; disease progression seems determined mainly by changing cytokine profiles in which the importance of certain key cytokines varies in the course of the disease process, in which inhibitors may play an important role [6]. In addition, other T cell responses have been identified, such as regulatory T cells and Th17 cells that have been shown to be implicated in various forms of leishmaniasis [7, 8].

### VL

In VL parasites can be found in all organs but particularly in the reticulo-endothelial system; aspirates of lymph node, bone marrow and spleen are used to demonstrate parasites in VL diagnosis.

#### Before treatment

After infection in the skin by the sand fly, the innate immune response includes interaction with neutrophils and complement after which the parasite infects macrophages/monocytes. Survival of the parasites is dependent on inhibition of macrophage activation and avoiding interaction with T cells [9].

Studies have shown that there is no cellular immune response against *Leishmania* parasites: peripheral blood mononuclear cells (PBMC) do not proliferate in response to stimulation with leishmanial parasites; in vivo the LST is typically negative [10]. This was confirmed in a later study by measuring cytokines in whole blood assays [11]. The cytokine profile in the blood has characteristics of a (anti-inflammatory) Th2 response and includes IL-6, IL-21 and IL-27 as well as transforming growth factor beta (TGF-β), sCD14 and tumour necrosis factor alpha (TNF-α) [12]. IL-6 is associated with lethality in VL caused by *L. infantum* [12]. There is a predominant role of IL-10 and TGF-β that correlate with parasite load [13–15]. However, during VL also high levels of IL-12 and interferon-γ (INF-γ) can be demonstrated that are typically associated with a (pro-inflammatory) healing Th1 response suggesting that there is no Th1 defect in active VL. The presence of an inhibitory factor such as IL-10 may be an important marker; blockade of IL-10 in ex vivo assays results in increased IFN-γ and TNF-α with reduced parasite growth [11, 16]. The complexity of the immune response cannot be captured in a Th1 and Th2 dichotomy alone [16, 17]. Other T cells that are implicated in VL include T regulatory cells (CD4^+^ CD25 ^+^FoxP3 ^+^ T regs) that play an important role and are correlated with high parasite load and production of IL-10 and TGF-β [15]. The role of Th17 cells is also important; [18] IL- 17 and IL- 22 that are effectors of innate immunity, are produced by Th17 cells and are thought to play a complimentary role to Th1 cytokines and inhibit parasite growth. This was found to be associated with resistance to VL, whereas a defect in Th17 responses may increase the risk of VL [19]. The role of CD8^+^ T cells is not clear; they may differentiate into effector cells that may kill target cells such as cytotoxic T lymphocytes that also produce TNF and IFN-γ. Other CD8 cells are memory T cells that may play a role in protection. Lastly, others acquire a regulatory role and are thought to play a role in prevention of immunopathology [20, 21]. There is a strong humoral response as evidenced by polyclonal gammaglobulinaemia; this consists of antileishmanial antibodies that are used in diagnosis such as in the direct agglutination test (DAT) and the rK39 ELISA. This humoral response may be stronger in Asia compared to Africa; the rapid diagnostic test (RDT) based on detecting rK39 antibodies has excellent sensitivity in Asia but is less useful in Africa because of lower levels of antibodies [22]. Other antibodies among the gammaglobulins include antibodies that can be found in connective tissue diseases such as SLE; this is thought to be the result of cross-reaction between leishmanial antigens and ribonucleotide antigens [23].

#### After treatment

After treatment of VL with antileishmanial drugs, the immune response changes to a predominantly Th1 response characterized by the presence of IL-12 production by antigen presenting cells and INF-γ production by T cells [24, 25]. Macrophage activation with subsequent killing of parasites is the key factor [19]. In addition to killing of parasites, commonly used antileishmanial drugs have immunomodulatory properties. Reduction of IL-10 and TGF-β occurs after treatment with stibogluconate (SSG), but this effect is stronger in patients treated with AmBisome or Fungisome, a locally produced liposomal amphotericin B from India, that causes down regulation of IL-10, TGF-β and IL-12 [26]. Miltefosine stimulates T cells and macrophages in vitro; in contrast to SSG, this was found to be non T-cell dependent in the mouse model [27, 28]. In vivo the LST becomes positive 6 months after treatment in 80 % of patients and this is thought to be a marker of immunity [2]. While from a clinical point of view this immunity seems permanent, the LST may revert back to negative [29]. It is not clear to what extent repeated exposure has a booster effect. In addition, any condition that decreases immune responses such as HIV infection, diseases that cause immunosuppression (e.g. haematological malignancies) or immunosuppressive therapy (steroids, immunomodulators), may be a risk factor for primary VL or a relapse of a previous VL episode as sterile cure probably does not exist. The humoral response gradually decreases but antibodies may persist for months and this makes serological tests less useful for monitoring disease outcome or predict relapse [30].

### PKDL

PKDL is characterized by an intermediate position between a Th2 and Th1 response and this translates into the typical clinical features. As a result of antileishmanial treatment of VL, the PKDL patient is no longer systemically ill, has no fever and the liver and spleen are no longer enlarged. Only in the skin parasites persist that may have been there since VL [31]. The immune response in PKDL is thus (partly) characterized by immune (re-)constitution, while at the same time there is dissociation of the immune response between the skin and the viscera. This causes the typical skin rash in the absence of systemic illness [1].

In PKDL the Th2 response shows the presence and persistence of IL-10 in the skin that was already present during VL, while systemically the Th1 response that was induced after VL therapy persists with IFN-γ production.[32–34]. In the skin, as typically the PKDL lesions occur in sun exposed areas of the skin (face, neck), the immune response is thought to be related to the influence of UV light; this causes damage to dendritic cells resulting in a Th2 type of response by inhibition of regulatory T cells. [32, 35–37] In PKDL increased levels of IL-10 expressing CD3+CD8+ regulatory cells were found in the skin that decrease after treatment [7]. Increased numbers of circulating CD8 lymphocytes showed impaired proliferation that was restored after treatment. This suggests that while overall in PKDL the CMI is intact (after stimulation with phytohaemagglutinin or *Leishmania donovani* antigen) and similar to healthy controls and cured VL patients, subtle differences exist [7, 35]. Other cells also produce cytokines and TGF –β, TNF-α, IL-10 and IL-12 produced by keratinocytes play a major role. While TNF-α levels are high, both in PKDL and VL low levels of TNF-α receptor 1 (TNFR1) were found, possibly caused by interference of high levels of IL-6 [38]. The ratio of TNF-α (inflammatory): IL-10 (anti-inflammatory) message was 2.66 and 1.18 in PKDL (skin biopsies) and VL (bone marrow aspirates), respectively, showing the importance of the dynamics of the cytokine profiles in various disease manifestations [38].

Macrophages are polarized to an M2 type (alternative activation) that is associated with suppression of cell mediated immunity and disease chronicity. Raised vitamin 1α,25 dihydroxyvitamin D_3_ levels possibly produced by the sun exposed skin in PKDL may play a role in the abnormal macrophage differentiation [39]. Matrix metalloproteinases (MMP) that are induced by TNF-α are involved in leucocyte recruitment and tissue remodeling may be important as well as inhibitors thereof (TIMP tissue inhibitor of matrix metalloproteinase) that are induced by IL-10. Serum levels of MMP9 and the ratio of MMP9 to TMIP1 (MMP9/TMIP1) are raised in active PKDL and return to normal in healed PKDL patients [38, 40]. TMIP3 is significantly elevated in PKDL compared to VL and is suggested to play a role in confinement of parasites to the skin in PKDL [38].

Th17 responses cause up regulation of Th17 markers such as IL-17, which is an inducer of TNF-α and NO levels in the PKDL lesion and in the blood; this is reversed by chemotherapy [41]. The differentiation into IL-17 secreting Th17 cells is induced by IL-23 as well as IL-1, IL-6 and TGF-β [41].

In human PKDL, miltefosine modulates the cytokine response by increasing levels of pro-inflammatory cytokines and decreasing anti-inflammatory cytokines; macrophages are activated [27, 28].

From a clinical point of view, the immune response that may vary over time and may differ between patients, dictates the clinical manifestation. The strength of the cell mediated immune response (CMI) is higher in acute PKDL, whereas chronic PKDL is associated with a weaker response [42]. Similarly, in macular PKDL the CMI is strong with few parasites and low antibody levels (only Ig1 is elevated), while in the polymorphic form (papulo-nodular) the CMI is low, induced by TGF- β and IL-10, with higher levels of markers for regulatory T cells, more parasites and high antibody levels, including both Ig1 and Ig3 (markers for IL-10) [36, 42–44]. Recently, immune complexes that were identified by PEG ELISA differed in quality and quantity between VL and PKDL with an association between IgG1 containing immune complexes found in VL and increased risk of subsequent PKDL [45].

**Fig. 3:**
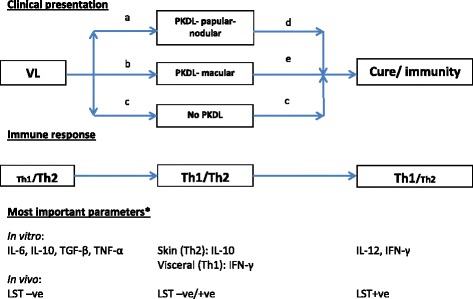
Schematic representation of clinical presentation, corresponding immune responses and areas of missing information using the Th1/Th2 dichotomy. *Most important parameters that determine the immunologically relevant response. *a, b* the intervals differ in time between regions: 0–13 months in Africa; 0–3 years in Asia. There is limited information on (the timing of) the developing or changing immune response in relation to type of VL treatment (or in case of absent VL history) and the clinical response (macular, papular, nodular PKDL; distribution and severity). *c* the interval after successful treatment of VL and establishment of permanent immunity is unknown. *d, e* there is no information on (the timing of) the developing or changing immune response (after PKDL treatment or after self-healing), the clinical response (reduction or disappearance of lesions) and development of immunity

Clearly the immunological changes described are dynamic and these have not yet been captured fully in available reports. There are no data that accurately describe the interval between VL and the development of PKDL (or absence thereof) in terms of immune responses, taking into account various parameters that may influence this. While in Sudanese PKDL the immune responses between VL and PKDL may form a continuum as the interval is short, this is difficult to understand in Asian PKDL in whom the interval may be 1–3 years or longer. It is not clear what happens between VL and PKDL and whether for example a predominantly Th1 response may revert to a Th2 type of response and why this would happen (Fig. 3). The possibility of a re-infection in a (partially) immune treated VL patient should be considered.

## Risk factors for PKDL in relation to immune responses

### Drug treatment for VL

In a study from Sudan a PKDL rate of 69 % was found in those treated with erratic doses of sodium stibogluconate (SSG) often of doubtful quality, whereas in those who received supervised treatment (SSG 20 mg/kg for 15 days) the PKDL rate was only 35 % [46]. Short course and high dose regimens seem to lower the PKDL rate as was shown for paromomycin [47]. In India, resistance for stibogluconate is common and PKDL rates decreased after introduction of amphotericin B as first line treatment for VL [43, 48]. These studies indicate that adequate treatment is important not only for killing of parasites but also for the development of an adequate immune response.

PKDL has been reported after VL treatment with stibogluconate, ambisome, miltefosine and paromomycin, but the exact PKDL rate after each drug is unknown as there are no studies with active follow-up [49–52]. In India, in a retrospective study of VL patients treated with AmBisome 20 mg/kg total dose, the cure rate for VL was 99.3 % with a 0.3 % PKDL rate [51]. In Bangladesh, a total dose of 15 mg/kg AmBisome for VL resulted in a PKDL rate of 10 % in a prospective study (Koert Ritmeijer, MSF-Holland, personal communication). In the South-East Asia region kala-azar elimination programme, single dose ambisome (SDA, 10 mg/kg) is one of the pillars and has excellent cure rates for VL of > 95 %; the PKDL rate is not known [53], (www.searo.who.int/entity/vector_borne_tropical_diseases/documents/SEA-CD-239/en/). In VL, a single dose of ambisome leads to lower parasite clearance compared with multiple dosing, but it is currently unclear whether PKDL rates are significantly lower in those with longer treatment duration [43, 54]. There is no information about combination treatment.

### Parasites

PKDL seems restricted to patients infected with *L. donovani* and this corresponds with the main epidemiological distribution in India and Africa. It should be noted, however, that the strains of *L. donovani* are completely different in these two regions from a molecular point of view [55, 56]. No evidence exists that directly links parasites in various regions to features of PKDL. It should be noted, however, that parasites that have developed resistance to e.g. stibogluconate (Sb) lead to poor outcome of VL and higher PKDL rate, probably mediated by a poor immune response. This was demonstrated in India, when changing from stibogluconate to amphotericin in the treatment of VL this seemed to reduce the occurrence of PKDL [48]. In vitro experiments show that Sb-resistant strains produce higher levels of IL-10 and TGF-β after interacting with PBMCs and block SAG-induced dendritic cell activation/ maturation; as these findings may also play a role in the pathophysiology of PKDL, further studies seem warranted [57, 58].

PKDL is rarely reported after VL caused by *L. infantum* or *L. tropica* infection unless there is co-infection with HIV [59, 60].

Although molecular analysis has shown differences between *L. donovani* strains in VL and PKDL probably as a result of antileishmanial drug treatment, they seem to be similar in terms of pathogenicity. PKDL strains keep their visceralizing ability in mice, providing support for the immune response as the main determinant for clinical manifestations (PKDL) [61–63].

### Genetics

Genetic factors may be responsible for the differences between endemic regions with regard to clinical and epidemiological findings. This was shown for the decreased function of the interferon-gamma receptor 1 gene (*IFNGR1*) that was found linked to development of PKDL in Sudan; this was not found for VL [64]. This was also detected in PKDL skin biopsies with uniform low expression of IFN-γ and *IFNGR1*, possibly explaining the persistence of parasites. Similar downregulation of the IFN-γ receptor was found in biopsies in a study from India [38, 65].

So far there has been no evidence for polymorphisms in the *IL 10* gene promotor [65].

## VL and PKDL and immunosuppression

Leishmaniasis is not uncommon in patients who are immunosuppressed. HIV co-infection is the most common cause; other conditions include patients with organ transplants or who are on immunosuppressive or immunomodulatory therapy, including the use of topical steroids [66, 67]. PKDL with concomitant HIV infection is more common and more severe [68]. All patients reported with PKDL and HIV co-infection had a CD4 count of less than 350 cells/mm^3^; in 95 % this count was less than 200 cells/ mm^3^ [59]. PKDL is no longer restricted to *L. donovani* and has been described in VL caused by *L. infantum* and *L. tropica*. Up to 30 % of HIV-VL co-infected patients have skin lesions resembling PKDL lesions but are more pronounced. Nodular lesions are common and florid and more extensive with unusual distribution; the acra are commonly involved. Hyperpigmentation and scaling may occur. Numerous parasites can be demonstrated in aspirates. Lesions may resemble Kaposi’s sarcoma or psoriasis; others may occur in pre-existing dermal conditions such as dermatofibroma, Kaposi’s sarcoma or even tattoos. HIV co-infected VL or PKDL patients could play a major role in transmission because of the high parasite load in the skin, the viscera and the blood [59]. The lesions may precede, coincide with or follow VL [59]. It is not always clear if PKDL follows VL or presents concomitantly with visceral disease (para kala-azar dermal leishmaniasis) or whether PKDL has regressed to VL [59]. In HIV co-infection the delineation between VL, PKDL and (disseminated) cutaneous leishmaniasis e.g. caused by *L. major* or *L. tropica* becomes blurred as cutaneous leishmaniasis may visceralize [59].

HIV infection and leishmaniasis both target macrophages and dendritic cells; HIV targets CD4 cells directly while *Leishmania* does this indirectly by promoting HIV replication in CD4 cells that play a pivotal role in the immune response needed to combat both infections; this leads to a predominant and aggravated Th2 immune response. In VL clinically this translates into a state of profound immune suppression with high parasitaemia and unusual clinical presentations. In HIV-VL co-infection the Th2 response that is characteristic of VL is exaggerated with decreased levels of IL-12 and IL-18 and interferon-γ, which is the adequate milieu for PKDL [69]. The Th17 cells subset response is lost earlier than that of Th1 cells [70].

Co-infected VL or PKDL patients are treated simultaneously with antileishmanial and antiretroviral therapy (ART). In some patients PKDL seems to occur in the context of immune (re)constitution as a result of ART, although it may have resulted from antileishmanial therapy only as both treatments may lead to immune (re-)constitution. Management is compounded by high recurrence rates. In some patients the CD4 cell count remains low despite successful antiretroviral therapy with undetectable viral load. AmBisome is the drug of choice for treatment of VL while intermittent AmBisome, stibogluconate or pentamidine have been considered as maintenance therapy, e.g. every 3–4 weeks [71].

## Immune manipulation in PKDL

Because of the incomplete immune response, combined antileishmanial therapy and immunomodulation has been studied. The autoclaved *L. major* vaccine did not prove to have sufficient immunogenicity to prevent CL and VL, in studies in Iran and Sudan, respectively [72, 73]. A next generation alum-precipitated autoclaved *L. major* vaccine was shown to be more immunogenic in healthy volunteers and was studied in chronic PKDL cases in Sudan who failed previous treatment with stibogluconate [74]. In those who were treated with SSG and vaccine, the cure rate after 6 months was 86 % while for those treated with SSG only, this was 53 % [75].

The rationale for immune manipulation is clear from a pathophysiological point of view. Currently prolonged courses with stibogluconate or miltefosine are given mainly aiming at killing of parasites and these drugs also have immunomodulatory effects. While in patients with chronic or disfiguring PKDL the indication for treatment is clear, in those with milder lesions any drug needs to have a good safety profile as the patients are not systemically ill or at risk of dying; in addition in Africa (Sudan) most of the patients will self-heal in 12 months. A safe immunomodulatory agent would target the immunological block and push towards a Th1 response. It could be argued that such an agent could also be added to VL treatment to achieve the Th1 response quickly so as to prevent PKDL from occurring. Currently, it seems that in the Indian subcontinent PKDL rates do not drop below 10 % despite VL therapy with excellent VL cure rates making combined immunochemotherapy an attractive option. Anti-IL-10 signaling blockade with monoclonal antibody is a candidate that was found to improve parasite-specific IFN-γ in patients with VL [76]. Further studies with similar compounds are expected.

## Conclusion

PKDL is an immunologically mediated condition and further understanding of the immune responses at various stages of clinical VL and PKDL, as well as in cure and during asymptomatic intervals is essential. PKDL is not a static or uniform disease and each patient may be different in terms of clinical presentation, chronicity, underlying immunological parameters, tendency to self-heal or response to drug therapy. Factors regarding drug therapy used, co-infections and genetics may therefore all influence the epidemiological and clinical features in various endemic areas.
